# Sex differences in disease: sex chromosome and immunity

**DOI:** 10.1186/s12967-024-05990-2

**Published:** 2024-12-27

**Authors:** Zuxi Feng, Minjing Liao, Liansheng Zhang

**Affiliations:** 1 The Second Hospital and Clinical Medical School, Lanzhou University, Lanzhou, 730000 China; 2 Department of Hematology, Lanzhou University Second Hospital, Lanzhou, 730000 China; 3 Gansu Province Clinical Medical Research Center for Blood Diseases, Lanzhou, 730000 China

**Keywords:** Sex differences, Sex chromosome, Immunity, X chromosome inactivation, Loss of Y chromosome

## Abstract

Sex is a fundamental biological variable that influences immune system function, with sex chromosomes (X and Y) playing a central role in these differences. Despite substantial evidence of disparities in immune responses between males and females, biomedical research has historically overlooked sex as a critical factor. This oversight has contributed to the observed disparities in susceptibility to autoimmune diseases, infectious diseases, and malignancies between the sexes. In this review, we address the phenomena and mechanisms through which aberrant expression of sex chromosome-linked genes contributes to sex-based differences in immune responses. We specifically focus on the implications of X chromosome inactivation (XCI) escape and loss of Y chromosome (LOY). Our review aims to elucidate the molecular mechanisms driving these sex-based differences, with particular emphasis on the interactions between sex chromosome genes and immune cells in both males and females. Additionally, we discuss the potential impact of these differences on disease susceptibility and identify prospective therapeutic targets. As personalized and precision medicine advances, it is crucial to integrate sex differences into immunological research and clinical trials. We advocate for an increased focus on sex-based considerations in fundamental, translational, and clinical research to promote personalized, sex-specific healthcare.

**Supplementary Information:**

The online version contains supplementary material available at 10.1186/s12967-024-05990-2.

## Introduction

**Fig. 1:**
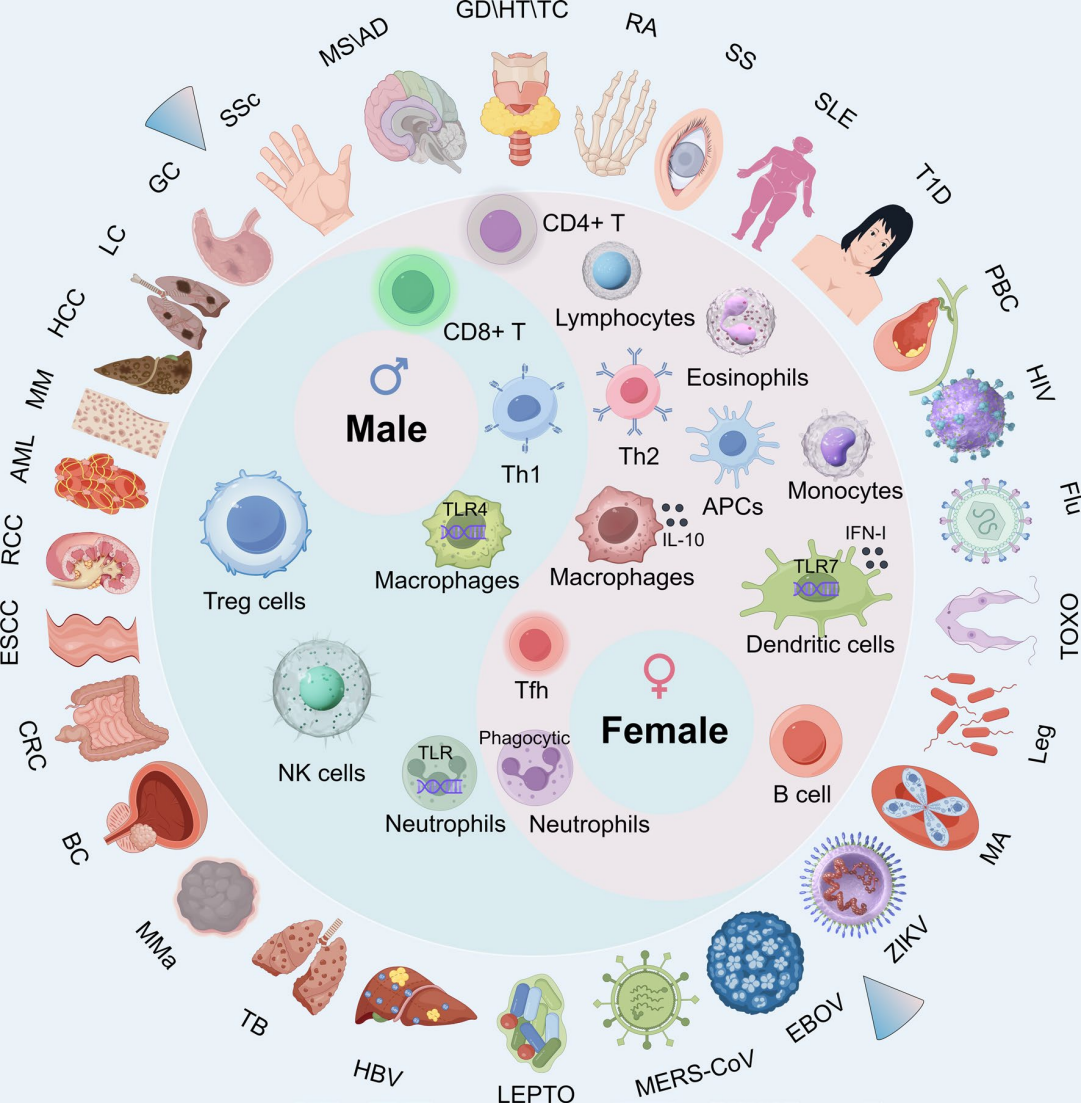
Summary of sex differences in diseases and immune responses. Lists of diseases with male-biased and female-biased incidence rates, and immune cells with male-biased and female-biased immune responses. *MS* multiple sclerosis, *AD* Alzheimer’s disease, *GD* Graves’ disease, *HT* hypothyroidism, *TC* thyroid cancer, *RA* rheumatoid arthritis, *SS* Sjögren’s syndrome, *SLE* systemic lupus erythematosus, *T1D* type 1 diabetes, *PBC* primary biliary cholangitis, *HIV* human immunodeficiency virus, *Flu* influenza, *TOXO* toxoplasmosis, *Leg* Legionnaires’ disease, *MA* malarial anemia, *ZIKV* Zika virus, *EBOV* Ebola Virus, *MERS-CoV* Middle East Respiratory Syndrome Coronavirus, *LEPTO* leptospirosis, *HBV* Hepatitis B Virus, *TB* tuberculosis, *MMa* malignant melanoma, *BC* bladder cancer, *CRC* colorectal Cancer; ESCC, Esophageal Squamous Cell Carcinoma; RCC, Renal Cell Carcinomap; AML, Acute Myeloid Leukemia; MM, Multiple Myeloma; HCC, Hepatocellular Carcinoma; LC, Lung Cancer; GC, Gastric Cancer

Sex chromosomes arose from an initially identical pair of autosomes, and the suppression of recombination has been a key driver in the evolutionary development of mammalian sex chromosomes. The X chromosome contains a multitude of protein-coding and non-coding genes, whereas the Y chromosome possesses comparatively fewer genes. The X chromosome is not only larger and more complex than the Y chromosome but also harbors a substantial number of genes associated with immune functions [1]. There are substantial distinctions between male and female immune systems, primarily influenced by genetic factors [2]. Recently, increasing numbers of researchers have been independently investigating the differences in immune responses and disease between sexes (Fig. 1). However, the molecular mechanisms by which sex chromosomes drive immune response differences between sexes, leading to disease-related sex differences, have yet to be fully studied and elucidated.

Sex differences in immune system responses are becoming increasingly evident, influencing the onset, symptoms, progression, and treatment outcomes of various diseases (Fig. 1). Females exhibit higher susceptibility to autoimmune diseases such as Sjögren syndrome (SS), systemic lupus erythematosus (SLE), hypothyroidism (HT), rheumatoid arthritis (RA), Graves' disease (GD), and other autoimmune disorders (Fig. 1). In contrast, males show higher incidence rates for certain cancers, including colorectal cancer (CRC), esophageal squamous cell carcinoma (ESCC), renal cell carcinomap (RCC), lung cancers (LC), and other cancers (Fig. 1). In infectious diseases, males are generally more susceptible to infections caused by human immunodeficiency virus (HIV), influenza, malarial anemia (MA), and other infectious diseases (Fig. 1), and tend to experience more severe disease courses with higher mortality rates. These differences are thought to be closely linked to sex-related differences in immune responses, hormone levels, and immune cell functions. The proper functioning of the immune system depends on a finely tuned balance between opposing forces, much like the concept of yin and yang (Fig. 1). Immune cells interact in a coordinated and regulated manner to sustain immune activity while preventing self-damage. Each type of immune cell has a counterpart that helps maintain this delicate equilibrium. For example, T lymphocytes are divided into subsets like Th1 and Th2, where Th1 cells are primarily involved in initiating aggressive immune responses, while Th2 cells act to suppress or regulate these responses, ensuring that immune activation is appropriately controlled. Similarly, B lymphocytes are categorized into effector B cells, which mediate immune attacks, and regulatory B cells, which help modulate immune function. Other immune cells, such as NK cells, macrophages, and neutrophils, also exhibit complementary counterparts with either pro-inflammatory or regulatory roles (Fig. 1). This intricate balance of opposing forces is critical for immune homeostasis, preventing autoimmunity, and ensuring that immune responses are triggered only when necessary and tempered when excessive. Maintaining this immune equilibrium is key to protecting the body against pathogens without causing harm to its own tissues.

In mammals, phenotypic sex differences can be attributed to genetic variations between XX and XY individuals. Notably, X chromosome genes affect immune cell composition and function, exacerbating sex differences [3]. The differences in X chromosome dosage between sexes are predominantly mitigated through X chromosome inactivation (XCI). XCI is an epigenetic mechanism driven by the X inactive-specific transcript (XIST), which randomly selects and silences one X chromosome in female cells, suppressing the expression of most of its genes. This process ensures dosage compensation of X-linked genes between female (XX karyotype) and male (XY karyotype) cells, supporting normal female embryonic development and maintaining stability throughout life [4]. However, around 15% of the genes on the inactive X chromosome evade XCI and remain expressed, referred to as XCI escape genes [5]. Consequently, the expression of XCI escape genes in female immune cell subpopulations is generally significantly higher than in males [6]. Many XCI escape genes lack functional counterparts on the Y chromosome, and these genes sometimes confer specific physiological advantages to females [7]. In healthy human females, age-related and individually variable expression of XCI escape genes, which also varies by tissue and cell, may explain sex-specific traits, phenotypic variation in X-linked diseases, and clinical anomalies in individuals with atypical X chromosomes [8]. For example, recent studies indicate that almost two-thirds of X-linked immune system-related genes are expressed without sex bias across non-lymphatic tissues, while the remainder exhibit similar frequencies of male and female bias, highlighting the complexity of XCI escape regulation [9]. Studies using GTEx data revealed that sex-biased gene expression is widespread but highly tissue-specific, with 13,294 sex-biased genes identified across various tissues, including candidates for XCI escape and hormone-related regulation of autosomal genes [10]. Thus, the increased dosage and interindividual heterogeneity of XCI escape genes may be crucial factors contributing to sex differences in disease.

miRNAs serve as potent regulators across a wide array of physiological pathways and are implicated in various disease mechanisms. The X chromosome has the greatest concentration of miRNA sequences, endowing females with a richer repertoire of miRNA-mediated regulatory mechanisms compared to males. Specifically, the human X chromosome encodes 118 miRNAs, in stark contrast to only 4 known miRNAs on the Y chromosome, thereby potentially influencing sex-specific manifestations of diseases [11]. Notably, X-linked miRNAs play pivotal roles in governing cellular fate, underscoring the notion that female cells may possess more robust epigenetic mechanisms than their male counterparts. Moreover, the presence and activity of X-linked miRNAs underscore significant sex disparities in the occurrence and progression of cancers, including discernible variations in DNA methylation patterns between sexes [2]. In immune cells, miRNAs exert regulatory control over immune responses, cytokine production, and immune cell activation. Given the abundance of miRNAs on the X chromosome, females are expected to exhibit heightened levels of these molecules, potentially contributing to sex-specific variations in immune cell function, such as differences in vaccine responsiveness. Ultimately, a comprehensive evaluation of sex-specific differences linked to miRNAs and immune cells holds promise for improved patient stratification and personalized healthcare interventions.

The Y chromosome’s sparse gene content and predominantly inactive state have led to its characterization as a genetic wasteland [12]. Variations in the Y chromosome can impact both the quantity and function of immune cells, thereby influencing immune system efficacy and disease resistance in individuals. Additionally, the Y chromosome is crucial in regulating tissue- and cell-specific alternative gene splicing, which is particularly significant in immune cells [13]. Loss of the Y chromosome (LOY) refers to the partial or complete absence of the Y chromosome in male cells, commonly observed in blood cells and progressively accumulating with age. As a mosaic event, LOY is closely associated with shortened lifespan and increased incidence and mortality of various diseases, including cancer and cardiovascular disorders [14]. The phenomenon of LOY has been consistently neglected in research concerning male health. LOY induces significant alterations in the genomic transcriptome of human immune cells, impacting biological processes including cell migration, angiogenesis, and immune responses. The absence of the Y chromosome in immune cells not only highlights its newfound role in sexual dimorphism but also provides insights into understanding and potentially treating diseases associated with LOY [15]. The integrity of the Y chromosome closely correlates with the median survival time of patients, with LOY carriers surviving approximately 5.5 years less than non-LOY patients [16]. LOY is prevalent in specific cancers, particularly in male patients; its frequency in cancer tissues might indicate its presence in immune cells and possible contamination from peripheral blood cells. In male cell lines, LOY may lead to common dependencies on specific genes, indicating potential therapeutic targets [17]. Differences in sex chromosomes play a significant role in the higher cancer incidence rates observed in males compared to females, partly due to the escape of certain genes on the X chromosome from XCI. However, LOY and extreme loss are also critical factors contributing to these sex differences in cancer incidence rates.

Comprehensively studying the molecular mechanisms underlying immune response variations between males and females in both immunology and clinical trials is of profound scientific and clinical importance for managing diseases that exhibit sex-specific immune response disparities. This review explores the influence of sex chromosome gene expression on human sexually dimorphic immune mechanisms, including phenomena such as XCI escape, and LOY. We advocate for researchers and clinicians alike to prioritize sex considerations in immunological and clinical research endeavors, aiming to advance personalized and precision healthcare initiatives.

## XCI in immune cells

XCI is a crucial mechanism in early mammalian embryonic development, maintaining sex-based gene expression balance by randomly silencing one of the two X chromosomes cells of females. This process begins early in embryogenesis, whereby each cell in the female embryo randomly selects one of the X chromosomes for inactivation, leading to the silencing of the majority of genes on the selected chromosome [5]. In immune cells, the maintenance of XCI involves dynamic mechanisms that lead to the overexpression of numerous X-linked genes, which has implications for female-biased autoimmune diseases [18]. XCI influences complex human traits, exhibiting varying degrees of skewing in blood, fat, and skin tissues, while maintaining consistent expression patterns in immune cells [19]. The random XCI in female cells achieves a balance in dosage compensation, which persists through successive cell divisions in all female somatic cells [20]. In certain lymphocyte subsets, including B cells and T cells, regulatory elements responsible for XCI can be lost after activation, enabling these activated immune cells to bypass XCI. This phenomenon enhances immune cell functionality and adaptive responses, allowing them to effectively respond to immune challenges while maintaining normal immune function. Upon B cell activation, the repositioning of Xist RNA and heterochromatin modifications may predispose X-linked genes to reactivation, potentially disrupting the sex-specific balance of gene expression [21]. Evidence indicates varied mechanisms for XCI maintenance in lymphoid and myeloid immune cells, including differential patterns of Xist RNA expression in innate immune cells such as NK cells and DCs derived from myeloid and lymphoid lineages, though not in plasmacytoid dendritic cells (pDCs) [5]. Importantly, compromised dynamic repositioning of Xist RNA could lead to XCI escape, potentially heightening susceptibility to autoimmune diseases in females [22]. While the specific mechanisms remain unclear, the quantity of proteins interacting with Xist/Xist RNA in immune cells is crucial for maintaining XCI function, and their involvement in promoting abnormal escape mechanisms associated with female-biased immune diseases warrants further investigation.

In summary, immune cells might gain advantages from increased expression of specific X-linked immune genes, with different immune cell types potentially showing unique sex-specific expression patterns for these genes. The mechanisms that regulate XCI maintenance within the immune system raise many intriguing questions that warrant further investigation.

## X-linked immune-related genes

**Fig. 2:**
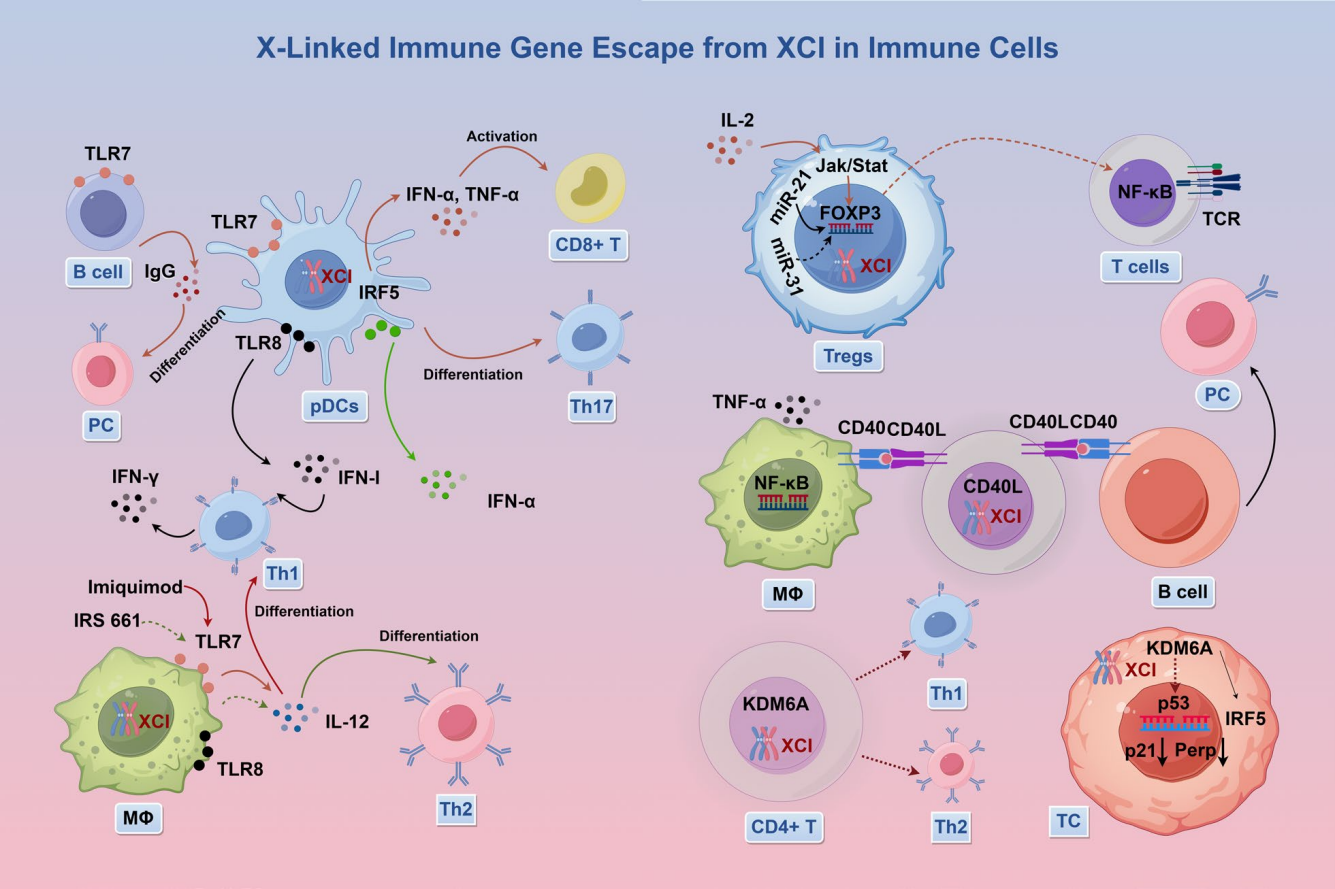
X-linked immune gene escape from XCI in immune cells. TLR7 and TLR8 escape XCI in human pDCs, affecting the body's immune response by regulating the functions of CD8 + T cells, Th1, Th17, and B cells. FOXP3 primarily regulates immune responses through Treg cells and T cells. CD40L modulates immune responses by regulating TNF-α secretion from macrophages. KDM6A can escape XCI in both CD4 + T cells and tumor cells

X-linked immune genes are vital for regulating immune cell functions, resulting in significant sex differences in immune responses. Recently, more XCI escape genes have been identified that affect immune cell recognition and pathogen responses, resulting in sex disparities in inflammation, antibody production, and immune memory (Fig. 2 and Table S1 in Supplementary material). Table S1 provides a comprehensive summary of these genes and their functions, including several underexplored genes that may hold significant potential in immune regulation. By compiling this information, we aim to present a thorough perspective on the mechanisms underlying sex differences in immune responses, offering valuable insights for further research in this field.

### TLR7

The TLR7 gene, located on Xq22.3, is a key mediator of immune responses. As an X chromosome escape gene, its expression in approximately 30% of immune cells in females enhances immune activity, contributing to sex differences in immune function [23, 24]. Elevated TLR7 expression in females results in greater IFN-α and TNF-α production in pDCs and stronger antibody responses in B cells compared to males. This heightened activity supports more efficient class switching, enhanced IgG production, and better plasma cell differentiation (Fig. 2) [25–28]. The increased TLR7 activity in females is associated with a higher prevalence of autoimmune diseases, such as SLE, and reduced efficacy of TLR7-targeted therapies in males. TLR7-mediated immune responses exhibit sex differences, with pDCs from females producing higher levels of IFN-α in response to HIV-1 infection, leading to stronger secondary activation of CD8 + T cells. This sex difference results in higher immune activation in females compared to males at the same viral load, potentially contributing to faster disease progression in women. Modulating the TLR7 pathway may therefore offer a novel approach to reducing HIV-1-associated pathology and improving sex-specific therapeutic strategies [29–32]. Studies have shown that pDCs in females exhibit a stronger TLR7/9-driven IFN-α response during viral infections, a sex difference that persists with age but diminishes after 60 years due to a decline in circulating pDCs [33]. These findings highlight the influence of X chromosome escape and sex hormones on sex-specific immune functions, as well as their implications for autoimmune disease susceptibility and immunotherapy efficacy. Future research should focus on developing TLR7-targeted therapies tailored to these sex-based differences.

### TLR8

The TLR8 gene, located on Xq24, is another X-linked gene known to escape XCI, contributing to significant sex differences in immune responses and disease susceptibility, including autoimmune disorders and cancers [34]. TLR8 signaling plays a pivotal role in immune regulation, particularly through enhancing Treg functionality and reprogramming glucose metabolism to boost anti-tumor immunity. This has been demonstrated in melanoma adoptive T cell transfer therapy models, where TLR8 activation improves Treg activity and strengthens anti-tumor immune responses [35]. In females, TLR8 expression from both X chromosomes is critical for diverse immune functions, including the production of IgG autoantibodies, upregulation of IFN-I, and the generation of neutrophils essential for robust immune responses (Fig. 2) [36]. In lupus-prone female mice, prolonged immune activation and elevated autoantibody production have been observed, implicating TLR8 in the pathogenesis of SLE. In these models, bone marrow-derived macrophages exhibit markedly increased TLR8 expression, correlating with heightened immune activity. Similarly, in patients with SSc, pDCs show elevated TLR8 expression and increased type I IFN activity, further supporting the association between TLR8 dysregulation and autoimmune disease progression [36]. The capacity of TLR8 to escape XCI and regulate key immune processes underscores its importance in sex-specific immune responses. Its involvement in autoimmune diseases like SLE and SSc, along with its impact on tumor immunity, highlights TLR8 as a promising target for developing sex-specific therapies. Future research should aim to further elucidate the mechanisms of TLR8’s escape from XCI in immune cells and its broader implications for disease susceptibility and immune modulation.

### CD40L

The CD40L gene, located on Xq26.3, is a key regulator of immune responses, primarily through its interaction with the CD40 receptor. Notably, CD40L escapes XCI in immune cells, contributing to sex-specific differences in immune function (Fig. 2) [37]. The CD40L-CD40 signaling pathway activates macrophages, promotes robust T cell responses, and stimulates B cell activation, proliferation, and differentiation into antibody-producing plasma cells [38, 39]. Additionally, CD40L expression on activated CD4 + T cells enhances antigen presentation and pro-inflammatory responses in dendritic cells, B cells, and macrophages [40]. The evasion of XCI by CD40L increases gene dosage in females, potentially contributing to their heightened susceptibility to autoimmune diseases. Overexpression of CD40L has been observed in healthy females and individuals with SLE, where it correlates with immune activation and disease progression [41]. In patients with X-linked hyper-IgM syndrome, mutations in the CD40L gene result in disrupted CD40/CD40L signaling, leading to abortive germinal center reactions, significant depletion, and phenotypical abnormalities of follicular dendritic cells, thereby impairing the functional development of B-cell follicles. Furthermore, persistent antigenic stimulation in mucosal tissues is likely a key factor in maintaining the elevated serum IgM levels observed in these patients [42]. Additionally, CD40L dysregulation is implicated in conditions like primary biliary cholangitis (PBC) and active SLE, where its upregulation leads to abnormal T and B cell activity. Monoclonal antibodies targeting CD40L have shown promise in reducing the production of anti-nuclear autoantibodies, suggesting therapeutic potential. In MS, CD40L activation on immune cells influences non-classical NF-κB signaling pathways, contributing to immune dysregulation. In RA, female animal models exhibit higher CD40L expression, particularly in arthritis models, correlating with stronger antigen-specific IgG responses and sex-specific immune differences. Increased frequencies of Tfh cells in females, producing IL-21 and IL-27, further promote sex-biased immune functions [43]. The overexpression of CD40L in females contributes to autoimmune disease susceptibility, while its dysregulation in conditions like SLE and MS highlights its potential as a therapeutic target. Monoclonal antibodies targeting CD40L may provide novel treatments for autoimmune diseases, and further studies are needed to elucidate its role in shaping sex-specific immune responses.

### FOXP3

The FOXP3 gene, located on Xp11.23, is a critical tumor suppressor that regulates immune system functions, particularly through its role in the development and function of Treg cells [44]. Its expression is tightly regulated by the IL-2/Jak/Stat signaling pathway, while its deficiency leads to severe immunopathology in both mice and humans [45]. Additionally, FOXP3 negatively regulates the NF-κB pathway, which is crucial for inhibiting T cell activation and maintaining immune tolerance. XCI significantly influences FOXP3 expression and its immune-regulatory functions (Fig. 2) [46]. Sex-based differences in FOXP3 expression contribute to disparities in immune system characteristics. In SSc, increased XCI skewing correlates with reduced FOXP3 expression, impairing Treg-mediated immunosuppression [47]. Conversely, in SLE, elevated frequencies of CD4 + FOXP3 + T cells strongly correlate with disease activity, emphasizing their role in limiting excessive immune responses [48]. In visceral adipose tissue, male mice exhibit higher frequencies of FOXP3 + Treg cells than females, with notable differences in phenotype, transcriptome, and chromatin accessibility [49]. These findings suggest that FOXP3 + Tregs play a key role in metabolic regulation and tissue-specific immune responses. Further exploration of these mechanisms may inform the development of targeted therapies for autoimmune diseases and cancer.

### CXCR3

The CXCR3 gene, located on Xq13.1, plays a crucial role in adaptive immunity and exhibits variable escape from XCI [50]. CXCR3 is a G protein-coupled receptor expressed on activated T cells, crucial for regulating T cell migration and function. It is activated by IFN-γ-inducible chemokines like CXCL9, CXCL10, and CXCL11, which recruit immune cells to inflammation sites and influence angiogenesis [51]. In females, CXCR3 leads to increased protein production, enhancing pro-inflammatory cytokine secretion and amplifying Th1 responses. CXCR3 expression is upregulated during inflammation on macrophages, dendritic cells, and both CD8 + and CD4 + T cells, facilitating their migration to infection sites [52]. CXCR3 modulates immune cell function through key signaling pathways, including JAK/STAT, PI3K/Akt, and Ras/ERK, which are critical for immune responses to exogenous antigens. These pathways suggest that CXCR3 may be targeted in immunotherapeutic strategies, particularly in organ transplantation [53]. TIn human clinical trials, CXCL10, a ligand for CXCR3, has been linked to enhanced vaccine responses, with elevated blood levels associated with increased antibody production [54]. In SLE, female patients show elevated CXCR3 expression in CD4 + T cells, correlating with demethylation of the CXCR3 promoter and increased disease activity. This overexpression is potentially due to the inactive X chromosome. While the direct escape of CXCR3 from XCI in human T cells remains unclear, increased expression in CD4 + T cells from SLE patients supports this hypothesis [55]. In contrast, reduced methylation of the CXCR3 promoter in CD4 + T cells leads to heightened CXCR3 expression, highlighting its role in PBC progression [56]. These findings underscore the significance of CXCR3 in adaptive immunity and disease progression. Its sex-specific expression and regulation via XCI and methylation offer potential for targeted immunotherapies to address sex-based differences in immune responses.

### KDM6A

The KDM6A gene, located on Xp11.3, encodes a lysine demethylase to regulate chromatin accessibility and transcriptional activity. Notably, KDM6A escapes XCI in both human and murine immune cells, leading to higher expression levels in female immune cells (Fig. 2) [57]. KDM6A deficiency lead to an increase in NK cell numbers and activity by influencing chromatin accessibility and gene expression, but impair their effector functions, potentially heightening susceptibility to viral infections [58]. In male glioblastoma (GBM) patients, KDM6A-induced T cell exhaustion correlates with GBM progression and response to immune therapies [59]. Functional inhibition of KDM6A has been shown to suppress IFN-γ production in murine effector T cells. In melanoma patients, KDM6A expression levels correlate with survival outcomes, where higher expression in females is linked to increased lymphocyte infiltration and enhanced IFN-γ production. Escape of KDM6A from XCI in females confers protective benefits, whereas loss-of-function mutations in males are associated with an increased incidence of certain cancers. Notably, this sex disparity in cancer incidence highlights KDM6A’s role in protecting females from bladder cancer through X chromosome maintenance and epigenetic mechanisms [60]. The loss of KDM6A leads to decreased expression of p21 and Perp, increasing susceptibility to bladder cancer [61]. In pancreatic cancer, KDM6A deficiency enhances sensitivity to BET inhibitors, suggesting potential therapeutic benefits of personalized treatment strategies [62].

KDM6A is also implicated in inflammatory responses. RNA-seq analysis of 102 COVID-19 blood samples revealed elevated KDM6A levels in female ICU patients, with no significant sex differences observed in males [63]. KDM6A activates the IFN-γ pathway, facilitating immune-regulatory cell recruitment and enhancing anti-tumor immune responses. KDM6A expression leads to H3K27me3 demethylation and increased IRF5 levels, promoting a pro-inflammatory microglial cell phenotype [64]. Moreover, in female ankylosing spondylitis patients, inflammatory KDM6A expression in Th17 cells correlates with increased transcriptional ratios of immune-regulatory KDM5C, suggesting potential targets for T cell-driven therapies in autoimmune diseases [65]. Elevated KDM6A expression in female CD4 + T cells also contributes to the mitigation of experimental autoimmune encephalomyelitis (EAE), influencing sex-based susceptibility to autoimmune diseases such as MS. In summary, the heightened expression of KDM6A in female tissues is crucial for maintaining cellular homeostasis, plays a key role in X chromosome-mediated tumor suppression, and offers greater protection against specific cancers in females. Additionally, its role in autoimmunity and inflammatory diseases underscores its potential as a therapeutic target for improving sex-specific treatment strategies.

## X-linked immune-related miRNA

**Fig. 3:**
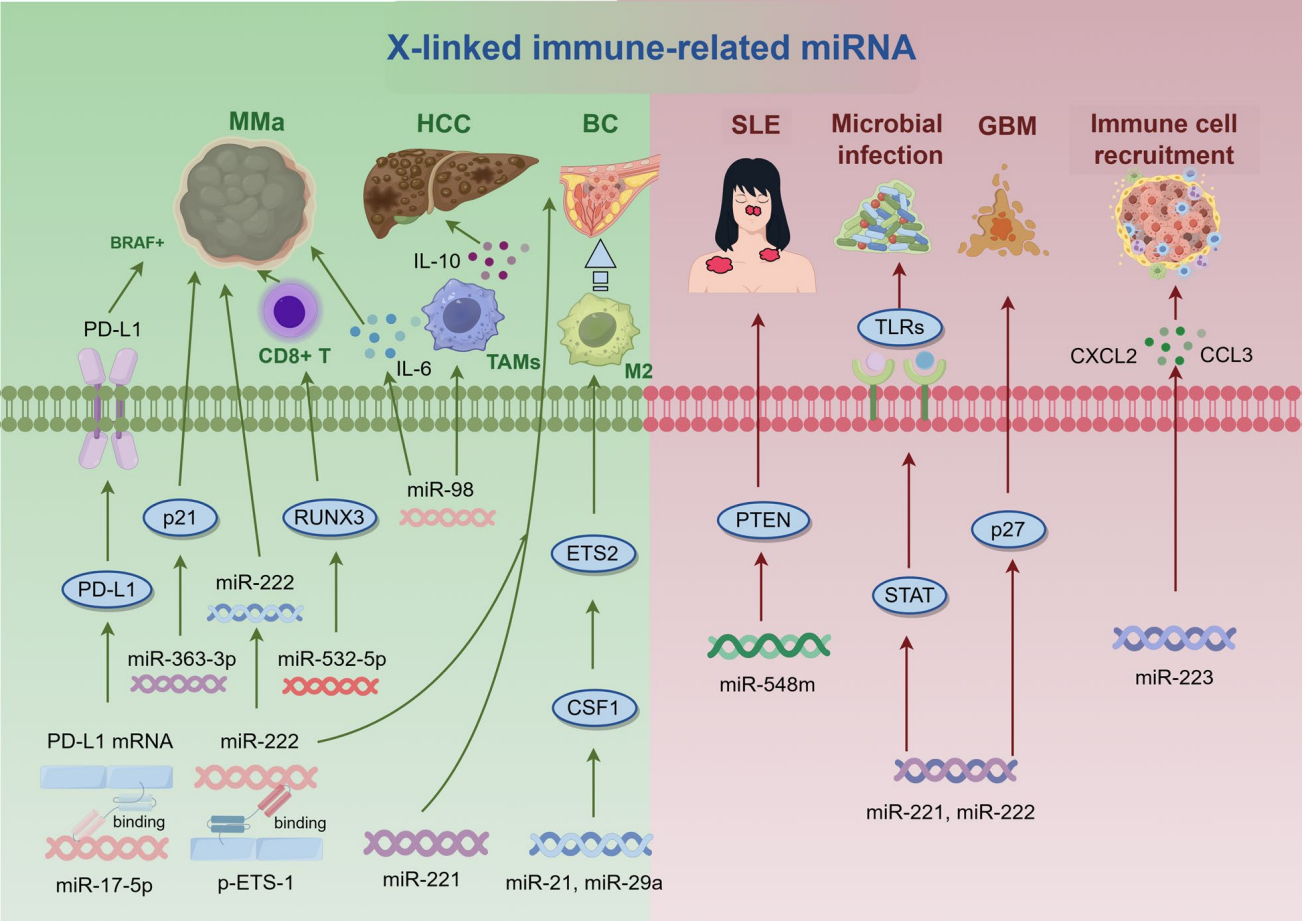
X-linked immune-related miRNAs. miR-17-5p regulates PD-L1 expression, affecting the invasion ability of drug-resistant cells. miR-363-3p inhibits melanoma cell cycle progression by targeting cyclin p21. miR-532-5p suppresses the expression of RUNX3. miR-21 and miR-29a promote tumor angiogenesis and proliferation by regulating CSF1-ETS2-M2 macrophage reprogramming. miR-548m participates in the regulation of SLE pathogenesis by targeting the PTEN gene. miR-221 and miR-222 help sustain low p27 levels, leading to ongoing cell proliferation in glioblastoma. Meanwhile, miR-223 limits the recruitment of innate immune cells by reducing the expression of CXCL2 and CCL3

miRNAs play essential roles in regulating both development and immune responses in the human immune system, displaying distinct sex-specific expression patterns that are modulated by environmental stimuli [66]. Notably, X-linked miRNAs play significant roles in sex-related immune responses by modulating gene expression. These X-linked miRNAs crucially impact immune cell function and development, disrupting immune tolerance and contributing to autoimmune disease pathogenesis [67]. Remarkably, about 50% of X chromosome miRNA expression is regulated by DNA methylation, with certain miRNAs evading XCI and exhibiting heightened expression levels in female cells [68]. X-linked miRNAs that escape XCI exhibit sex differences in various tumors, influencing immune cell infiltration and tumor invasiveness (Fig. 3) [69].

In melanoma, miR-17 regulates PD-L1 expression and influences drug-resistant cell invasiveness, thus impacting prognosis and treatment efficacy [69]. Sex bias in breast cancer involves 125 identified genes, predominantly affecting females (114 genes) versus males (11 genes). Among these, eight genes closely associated with immune cell infiltration highlight sex-specific influences in breast cancer progression [70]. miR-21 and miR-29a are crucial in M2 macrophage reprogramming through the CSF1-ETS2 pathway, promoting tumor angiogenesis and proliferation [71]. Additionally, miR-23a exhibits sex-specific effects in cerebral ischemia by targeting XIAP mRNA, altering cell death pathways differently in male and female mice [72]. In melanoma and hepatocellular carcinoma (HCC), miR-98 suppresses IL-6 and IL-10 expression, respectively, influencing tumor cell migration and invasion, thereby impacting patient survival [73]. Conversely, overexpression of miR-106a and miR-363 is associated with the oncogenesis of T-cell leukemia [74], while downregulation of miR-374b is associated with a poor prognosis in T-cell lymphoblastic lymphoma patients [75]. This correlation affects cell proliferation and apoptosis sensitivity through targeting of AKT1 and Wnt16 [75].

miR-221 and miR-222 are well-studied X-chromosome loci in tumors, widely distributed across eukaryotes [76]. In metastatic melanoma, miR-222's promoter binds directly to ETS-1, where phosphorylation status influences its expression; conversely, non-phosphorylated ETS-1 in early melanoma suppresses miR-222 transcription [77]. Furthermore, these microRNAs facilitate hematopoietic differentiation in human embryonic stem cells (HPSCs) by targeting the SCF/c-KIT axis, enhancing progenitor cell output and multipotency. Modulation of miR-221 and miR-222 levels in undifferentiated HPSCs alters c-KIT expression, promoting hematopoiesis and offering insights into leukemia pathogenesis [77]. miR-221 and miR-222 also regulate p27 levels in cancer cells, influencing sustained proliferation, notably in GBM [78]. They additionally modulate tolerance to microbial infections via TLR responses, affecting SWI/SNF and STAT pathways. Their reprogram macrophage function through chromatin remodeling, potentially impacting immune response and patient prognosis, highlighting their biomarker potential in sepsis [79]. miR-223, highly expressed in melanoma, reshapes tumor-infiltrating myeloid cells to support metastasis and angiogenesis, with sex differences possibly linked to X-chromosome inactivation [80]. miR-363-3p inhibits melanoma cell cycle progression by targeting p21, implicating it as a therapeutic target for cancer treatment [81]. In immune contexts, miR-374b and miR-532-5p exhibit dysregulation in melanoma, influencing pathways like Wnt-16, AKT1, and RUNX3, affecting CD8 + cell presence and tumor infiltration [82]. X-linked miRNAs, notably in active lupus, show significant sex-specific expression in CD4 + T cells, influenced by epigenetic mechanisms like demethylation, though comprehensive understanding of XCI-escaping miRNAs requires further investigation [55]. miR-548 m's regulation of PTEN affects SLE pathogenesis [83], while miR-548am-5p, identified as an XCI escape gene, demonstrates sex-specific apoptotic susceptibility modulation, highlighting its role in disease etiology [84]. Sex-biased miRNA expression plays a role in sex differences in disease outcomes, particularly in immune responses, highlighting the necessity for more comprehensive mechanistic investigations into X-linked miRNAs in immune cells [78].

miRNAs play a pivotal role in disease treatment, and various ongoing oncology-related clinical trials are investigating their applications in screening, diagnosis, and drug testing. The sex-specific expression of miRNAs offers valuable insights for future miRNA-based personalized therapeutic strategies. In breast cancer, altered expression of miR-221 and miR-222 has been closely linked to drug resistance, with miR-221 promoting doxorubicin resistance through the PTEN/Akt/mTOR signaling pathway, while miR-222 and miR-29a modulate therapeutic resistance by targeting PTEN [85, 86]. Preclinical studies and trials have demonstrated the therapeutic potential of second-generation miR-17 inhibitors, such as RGLS8429, and miR-29 mimics, such as MRG-229 [87–89]. Overexpression of miR-21 plays a critical role in chemotherapy and radiotherapy resistance across various cancers by downregulating tumor suppressors, including PTEN and PDCD4, thereby mediating resistance to drugs like paclitaxel and cisplatin [90, 91]. Combined therapeutic approaches targeting miR-21, such as co-delivery with 5-FU, have shown efficacy in overcoming resistance [92]. Similarly, miR-23a and miR-374b are implicated in radiotherapy and chemotherapy resistance in lung and hepatocellular cancers, respectively [93, 94]. miR-23a promotes radioresistance via angiogenesis, while miR-374b reverses sorafenib resistance by suppressing glycolytic pathways. Moreover, miR-221 inhibition significantly enhances the sensitivity of triple-negative breast cancer cells to paclitaxel, and low miR-223 expression is associated with resistance to dual antiplatelet therapy and an increased risk of cardiac events [95]. In summary, The diversity of miRNAs in therapeutic resistance mechanisms and their potential for targeted interventions, particularly when considering sex-specific differences in miRNA expression, strongly support the development of novel anti-cancer strategies tailored to personalized treatments.

## Loss of Y chromosome (LOY)

### LOY and immunity

**Fig. 4:**
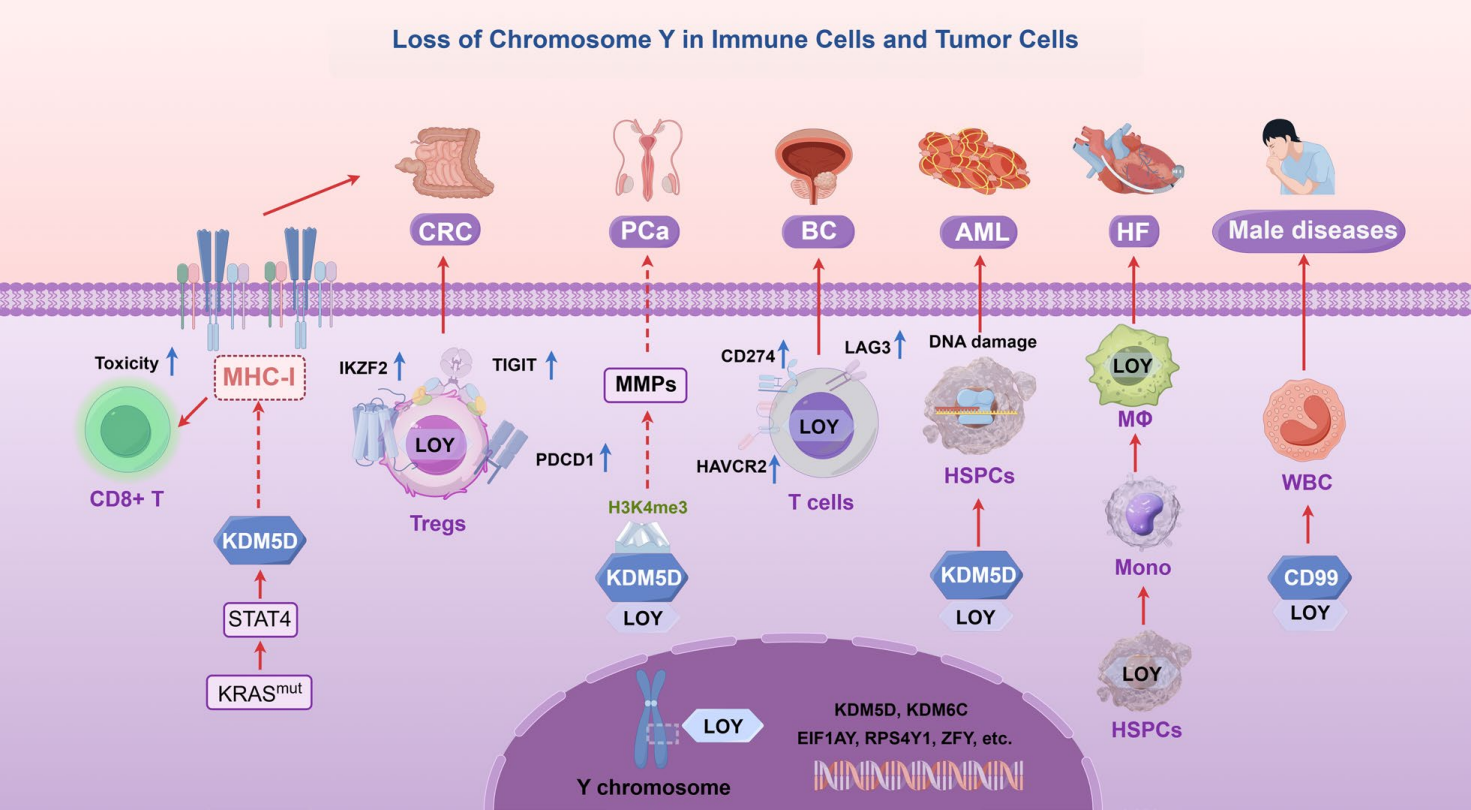
Loss of Y chromosome Y in immune cells and tumor cells. The upregulation of the Y-chromosome gene KDM5D significantly impacts tumor cell adhesion and tumor immune response through KRAS mutation-STAT4 mediation. KDM5D inhibits prostate cancer cell invasion by demethylating H3K4 and suppressing the expression of matrix metalloproteinases. In bladder cancer cells, LOY enhances the expression of immune checkpoint molecules such as CD274, LAG3, and HAVCR2. This upregulation contributes to T cell exhaustion and makes the cells more vulnerable to PD-1 immune checkpoint therapy. The deletion of KDM5D can result in clonal expansion of hematopoietic stem and progenitor cells (HSPCs) and progression of acute myeloid leukemia (AML). LOY macrophages excessively activate pro-fibrotic signaling pathways, which in turn stimulates fibroblast activation, proliferation, and the production of excessive extracellular matrix, ultimately resulting in myocardial fibrosis and heart failure

LOY in immune cells is linked to higher rates of early male mortality and a variety of diseases. Specifically, mosaic LOY (mLOY) in leukocytes correlates with heightened overall mortality risk in male, particularly in relation to cancer-related mortality. This discovery challenges previous assumptions that underestimated the potential consequences of LOY, underscoring the need for further investigation into this phenomenon [96]. Notably, the occurrence and gene expression alterations of LOY vary significantly across different immune cell types in patients with various diseases (Fig. 4). In a UK Biobank study involving 207,603 cancer-free male, 1.6% exhibited low levels of immune cell mLOY, while 0.3% exhibited high levels [97]. LOY predominantly affects various leukocyte types such as NK cells and CD4 + T cells, leading to transcriptional abnormalities in approximately 500 genes [98]. This supports the hypothesis that LOY in blood could facilitate disease progression by modifying immune cell functions that are affected by genetic mutations. Single-cell transcriptomic sequencing reveals varying degrees of LOY across different cell types: 27% in NK cells, 23% in monocytes, 7% in B lymphocytes, and 3% in T lymphocytes [98]. In mouse models, the Y chromosome significantly influences immune cell numbers and gene expression, particularly regulating NK T cell numbers, CD4 + T cell gene expression patterns, and macrophage immune responses. Studies demonstrate that male M1 macrophages exhibit significantly increased expression of pro-inflammatory genes during LDL uptake, whereas female macrophages tend to display markers associated with cellular damage [99]. The Y chromosome, which is rich in genes related to wound healing and suppression of immune responses, corresponds with the traits of anti-inflammatory M2-like tumor-associated macrophages, though macrophages can display various phenotypes beyond the M1 and M2 classifications [100]. Genes within the PAR1 region of the Y chromosome exhibit male-biased expression, whereas most non-PAR1 escape genes exhibit female-biased expression [101]. Whole-genome analyses of peripheral CD4 + T cells from healthy individuals reveal a higher accessibility of X-linked chromatin sites in female cells [102]. Despite its functional limitations, the Y chromosome plays an indispensable role in immune responses and reproductive systems, thereby underscoring its critical significance in human biology.

### LOY in cancer

With advancing age, the LOY in male cells is strongly correlated with the incidence of cancer [103]. Specifically, genes RBMY and TSPY located on the Y chromosome are aberrantly activated in male HCC, resulting in elevated TSPY expression and reduced TSPX expression in affected male patients. This dysregulation correlates with increased levels of the HCC marker glypican-3, while TSPX expression exhibits a negative correlation with glypican-3 and FOXM1 [104]. In CRC, certain male patients display feminization of cancer cells, a phenomenon notably linked to CpG island methylation seen in female CRC. Conversely, male CRC often presents feminization-like chromosomal copy number abnormalities such as X chromosome gains or LOY [105]. As male age, there is a notable decrease in Y chromosome presence in leukocytes [106]. LOY is prevalent in the tumor microenvironment of CRC, particularly within Treg cells, potentially enhancing their immunosuppressive properties. LOY correlates with elevated expression of immune checkpoint receptors like PDCD1, TIGIT, and IKZF2 in Treg cells, thereby influencing their function in the tumor microenvironment and potentially affecting immune therapy responses in CRC patients [107]. Recent studies underscore the role of mLOY in shaping immunosuppressive tumor microenvironments. In specific contexts, such as in macrophages, LOY has been implicated in promoting cardiac fibrosis and heart failure, whereas in cancer immunology, its presence in Treg cells is crucial for evading immune responses [108]. Male CRC patients are notably susceptible to metastasis and increased mortality, phenomena closely linked to Y chromosome alterations and immune cell dynamics. Notably, recent research has notably highlighted the upregulation of the Y chromosome gene KDM5D in KRAS-mutated CRC, which influences tumor cell adhesion and immune responses through the KRAS-STAT4 signaling pathway [109]. Specifically, KDM5D epigenetically suppresses tight junctions and downregulates MHC-I expression, facilitating immune evasion by tumors. Deletion of KDM5D conversely enhances tumor cell adhesion and improves CD8 + T cell-mediated tumor surveillance, enhancing immune response effectiveness [109]. Recent investigations emphasize the prevalence of LOY in Treg cells within the blood and tumor tissues of prostate cancer patients, surpassing levels found in other T cell subsets and contributing to immune dysregulation. LOY, identified as the most common somatic aneuploidy in aged male leukocytes, correlates with heightened overall mortality rates and increased cancer susceptibility due to LOY. The buildup of LOY in Treg cells may significantly impact immune function in prostate cancer contexts [110]. In metastatic prostate cancer, frequent deletion of the KDM5D gene predicts poor prognosis, limiting tumor invasiveness through H3K4 demethylation and suppression of matrix metalloproteinase expression [111]. Similarly, in the context of bladder cancer, LOY is linked to an invasive tumor phenotype and a worse prognosis. Mechanistically, bladder cancer cells with LOY induce dysfunctional or exhausted CD8 + T cells, impairing T cell function within the tumor microenvironment [112]. LOY is observed in ~ 23% of male bladder cancer patients across various tumor grades and stages, indicating its potential as an early event in bladder cancer pathogenesis [113]. Despite the aggressive growth of tumors associated with reduced Y chromosome gene expression in these instances, these tumors paradoxically exhibit improved responses to immunotherapy, particularly immune checkpoint blockade with PD-1/PD-L1 inhibitors [114]. Furthermore, LOY genes KDM6C and KDM5D correlates with adverse prognosis in bladder cancer patients, further compromising anti-tumor immunity within LOY-associated tumors [115]. This loss results in CD8 + T cell dysfunction, exacerbating tumor invasiveness [115]. In male head and neck squamous cell carcinoma (HNSCC), LOY is widespread, impacting ~ 25% of affected individuals and correlating with diminished cancer survival rates [116]. Recent findings also highlight widespread LOY in pancreatic cancer tissues of male patients, in stark contrast to intact Y chromosomes found in chronic pancreatitis tissues, suggesting LOY as a potential diagnostic marker distinguishing benign from malignant pancreatic diseases [117]. The presence of LOY in blood cells shows a strong correlation with the development of cancers in males, including an increased risk of clonal expansion in HSPCs and advancement to acute myeloid leukemia (AML) [118]. Studies using mouse models have demonstrated that LOY induces significant DNA damage in HSPCs, promoting clonal hematopoiesis and contributing to AML pathogenesis [118]. Notably, the KDM5D gene encodes the human H-Y antigen, which may lead to immune-mediated rejection of male organs and bone marrow transplants in female recipients, despite matching of the major histocompatibility complex [119]. The identification of the H-Y antigen holds potential for predicting post-transplant outcomes, aiding in prenatal diagnostics, and informing reproductive strategies [119].

### LOY in non-neoplastic diseases

In mouse models, young male mice demonstrate relative resistance to EAE, whereas susceptibility increases notably in elderly males and females. Age-related severity of EAE in males contrasts with stable conditions observed in females [120]. The introduction of exogenous Y chromosome exacerbates EAE severity, underscoring its pivotal role in sex disparities [120]. Gene expression in CD4 + T cells of male EAE patients mirrors patterns observed in Y chromosome-expressing mouse models [121]. Studies further indicate a significant LOY in male patients with abdominal aortic aneurysm, correlating with decreased SRY expression and free testosterone levels with advancing age [122]. Similarly, male PBC patients exhibit a pronounced LOY, which intensifies with age [122]. Furthermore, the Y chromosome correlates with T cell infiltration, particularly notable in stroke-prone hypertensive rats, suggesting potential roles in cardiovascular regulation via organ-specific T cell infiltration [123]. Male patients with autoimmune thyroiditis demonstrate a markedly elevated frequency of LOY [124]. Notably, with age, HSPCs in males experience LOY. Consequently, bone marrow-derived Y chromosome-deficient monocytes infiltrate the heart post-cardiac injury, differentiating into Y chromosome-deficient macrophages that replace existing cardiac macrophages [108]. Intriguingly, these overactive macrophages stimulate fibrosis signaling pathways, which in turn boost the activation, proliferation, and extracellular matrix production of fibroblasts. This process eventually results in myocardial fibrosis and contributes to heart failure [108]. Transplantation of Y chromosome-deficient bone marrow cells escalates animal mortality and age-related fibrotic lesions, particularly affecting cardiac function. Conversely, therapeutic neutralization of TGF-β1 shows promise in improving cardiac function in Y chromosome-deficient mice [125]. In COVID-19 patients, particularly males and the elderly, observed LOY correlates closely with higher mortality rates. LOY contributes to immune dysfunction, initially attenuating the immune response to SARS-CoV-2 and thereby increasing the risk of disease progression [126]. LOY predominantly impacts granulocytes and monocytes in patients with COVID-19, showing a positive correlation with disease severity, mortality during ICU treatment, and a history of vascular diseases [126]. Subsequent recovery stages exhibit significant reductions in LOY levels and low-density neutrophil counts, suggesting a role in male innate immune function [127]. Impaired immune cell function due to LOY compromises effective virus resistance. Additionally, LOY loss partly explains sex disparities and higher mortality rates in elderly male COVID-19 patients [126]. Genetic variations in the Y chromosome influence how male mice respond to influenza virus infection, enhancing the immune response while not affecting virus replication [128]. A study comparing sex-specific gene expression among neurons, astrocytes, and microglia in the brains of newborn mice revealed that microglia exhibited the highest degree of sex-specific gene expression, followed by neurons and astrocytes. These sex-specific genes are predominantly enriched in immune systems and immune-related diseases, with Y chromosome genes playing a dominant role in these cells. For instance, overexpression of Eif2s3y in male neurons increases synaptic transmission, leading to autism-like behavior in male mice [129]. Transcriptomic analysis of macrophages and CD4 + T cells reveals significant differences in Y-linked gene expression among susceptible mouse strains, findings corroborated by human studies [121]. Specific genetic variations in the male Y chromosome correlate with a heightened susceptibility to coronary artery disease, linked to immune and inflammatory molecular pathways, particularly in monocytes and macrophages. Y chromosome-regulated genes are notably enriched in immune response genes, crucially involved in antigen processing and presentation [130]. Changes in gene expression in specific regions of the Y chromosome in macrophages lead to downregulation of KDM6C and PRKY genes [131]. Reduced KDM6C expression in macrophages significantly decreases immune co-stimulatory signals, affecting 59 signaling pathways [132]. CD99 expression on Y chromosome PAR1 is significantly diminished at the transcript and protein levels across various immune cells. However, male LOY is associated with CD99 expression in immune cells, crucial for normal leukocyte transendothelial migration [133]. Reduced Y chromosome levels may hinder immune cell migration, impacting immune-related cellular functions and disease susceptibility in affected males at the protein level [133].

## Discussion and future direction

To date, notable sex disparities exist in the incidence, symptomatology, disease progression, and treatment outcomes across various diseases, including infectious diseases, autoimmune disorders, cardiovascular diseases, and cancers. Concurrently, sex disparities in immune responses persist and evolve, both in normal physiological conditions and disease states (Fig. 1). Sex differences in immune responses are complex and multifaceted, involving mechanisms such as immune cell activation, immune molecule expression, and hormonal regulation. The balancing role of the immune system can be compared to the throttle and brake of a car, where the throttle provides power and the brake prevents excessive reactions. The immune system does not need to be stronger in all aspects; rather, it must maintain a balance, or yin-yang equilibrium, that effectively clears pathogens without causing harm to the body’s own tissues (Fig. 1). Disruption of this balance may lead to either overactivation or insufficient activation of the immune system, resulting in autoimmune diseases or immune deficiencies. Therefore, maintaining immune balance is crucial for disease prevention and overall health. These gender differences not only enhance our understanding of diseases but also provide new perspectives for developing personalized treatment strategies. Further research into the immune mechanism differences between genders will help to formulate more precise and tailored clinical approaches, better addressing the needs of different gender groups. However, factors and mechanisms driving these immune response disparities between sexes, along with their implications for sex-specific disease outcomes, have received limited research attention and explanation. This review aims to elucidate known or potential molecular mechanisms through which sex chromosomes regulate immune functions, influencing observed differences in disease manifestations between sexes.. These mechanisms discussed include XCI escape and LOY.

Currently, a growing list of X-linked immune genes has been recognized for their ability to evade XCI, although they represent a minority among X-linked immune genes (Table S1 in Supplementary material). Recently, more XCI escape genes have been identified that are closely associated with immune cell recognition and pathogen responses, contributing to sex disparities in inflammation, antibody production, and immune memory. X chromosome-related genes (such as CD40L, FOXP3, CXCR3, and KDM6A) enhance immune responses in females by regulating immune cell functions, contributing to sex differences in autoimmune diseases and cancers (Fig. 2). Notably, XCI escape genes are more prevalent in autoimmune disorders, particularly SLE. This escape from XCI is particularly common in immune cells and is subject to dynamic regulation, potentially increasing the susceptibility of females to autoimmune conditions. Conversely, in cancer, the escape of oncogenic genes from XCI paradoxically appears protective in females to some extent. This raises intriguing questions regarding the causal link between X-linked gene escape from XCI and sex disparities in autoimmune disease susceptibility. Understanding the stability of XCI escape in immune cells and the molecular mechanisms behind it is crucial and requires further investigation. Furthermore, the unresolved heterogeneity in mechanisms facilitating X-linked gene escape warrants attention. Understanding the regulation of tissue-specific escape mechanisms and their impact on immune cell functionality is crucial for deciphering the high levels of X-linked genes in autoimmune diseases. Moreover, the maintenance of dosage compensation on the inactive X chromosome in immune cells without the presence of Xist RNA and heterochromatin marks remains an open inquiry. Some researchers speculate that DNA methylation might sustain transcriptional repression, but detailed mechanisms necessitate further elucidation [134]. Additionally, the role of XCI escape in immune cells in promoting sex-biased immune-mediated diseases remains obscure. Recent advancements in bioinformatics and genomics have continually refined methods for studying XCI processes. For example, Sauteraud et al. developed the XCIR tool, an R package for analyzing escape genes in RNA-seq data, facilitating the identification of genes undergoing XCI escape during XCI [135]. Recent Assay for Transposase-Accessible Chromatin with high-throughput sequencing (ATAC-seq) technologies have disclosed significant individual variations in the regulatory elements of CD4 + T cells, with sex emerging as a major source of variability. Specific regions on the X chromosome exhibit double the signal intensity in female cells compared to male cells, indicative of XCI escape gene. The widespread application of ATAC-seq has unveiled individual variability in gene regulatory elements on the X chromosome, offering new insights into the association between sex differences and disease occurrence [136]. Overall, much remains to be explored regarding X-linked immune gene escape from XCI, including its role in driving sex disparities in disease susceptibility and other pertinent aspects. Current experimental techniques for studying XCI escape are predominantly limited to allele-specific markers or RNA FISH analysis [28], with no standardized markers developed yet for efficient, rapid, and cost-effective identification of XCI escape.

miRNAs play a vital role in controlling immune gene expression by modulating the activation, proliferation, and differentiation of immune cells. However, many studies on miRNAs have overlooked sex-specific backgrounds. Altered miRNA expression patterns in immune cells are prominent in autoimmune diseases, which significantly influence disease incidence and prognosis [137]. X-linked immune-related miRNAs, which escape XCI, play crucial roles in immune responses and disease progression, showing sex-specific patterns in cancer and autoimmune diseases. These miRNAs, such as miR-21, miR-221, and miR-222, regulate immune cell function, tumor invasiveness, and therapeutic responses. Their involvement in pathways like AKT, Wnt, and TLR underscores their potential as biomarkers and therapeutic targets (Fig. 3). Notably, the functions of most X-linked miRNAs remain undescribed to date. Analyzing sex-specific miRNA expression heterogeneity can significantly deepen our insight into the mechanisms driving sex-biased diseases and provide new perspectives for developing sex-specific therapeutic strategies. The distribution of miRNAs on the X chromosome is closely linked to sex-related functions and pathological processes in immune responses and cancer, warranting further investigation to improve disease treatment and prevention [138]. X-linked miRNAs exhibit sex-specific immune differences, offering a protective mechanism in females, although controversies exist in males. Many X-linked miRNAs are still not well-studied in either sex. Investigating sex-biased expression of specific miRNAs could uncover sex-specific pathological conditions. Future research should address this gap to connect clinical data with the molecular mechanisms of sex-biased diseases, thereby facilitating the development of novel therapeutic approaches. In immune cells, X-linked miRNAs regulate critical processes such as differentiation, maturity, homeostasis, and function, which are indispensable for immune responses. Their dysregulation is linked to various diseases, including cancer, inflammation, and autoimmune disorders, offering potential new avenues for diagnostic markers and gene therapy methods [139].

While X-linked genes are well-studied in relation to sex differences in tumors, the role of Y-linked genes remains incompletely understood. Nevertheless, genetic material from the Y chromosome is predominantly expressed in males, potentially influencing adverse prognoses in male tumor patients [140]. LOY, a sex-specific genetic variation, correlates with various health issues, including cancers and cardiovascular diseases. However, many questions persist. LOY in immune cells is associated with higher mortality rates and increased susceptibility to various diseases, particularly cancer. Mosaic LOY (mLOY) in leukocytes is linked to transcriptional abnormalities in immune cells, such as NK cells and T cells, and affects genes related to immune function. LOY plays a crucial role in cancer progression by influencing immune responses in Treg cells and macrophages, potentially contributing to immune evasion. LOY also impacts non-neoplastic diseases like autoimmune conditions and cardiovascular diseases, suggesting its broad implications in health and disease (Fig. 4). The role of LOY in cancer cells in regulating T cell function and creating an immunosuppressive microenvironment, which accelerates cancer progression, is still unclear. Further research is necessary to better understand how LOY in cancer cells interacts with LOY in tumor-infiltrating immune cells throughout the course of cancer progression. The link between LOY and aggressive cancer traits, particularly in bladder cancer and CRC, remains contentious and requires further investigation. A major challenge is the precise identification of LOY in various clinical samples. Current techniques for detecting LOY include DNA microarrays, whole genome sequencing, and droplet digital PCR. Recent advancements include the use of GWAS-derived Y chromosome single nucleotide polymorphism data and tools like YTool for data analysis [141]. However, these methods are labor-intensive and lack adequate sensitivity and resolution. These limitations hinder a full understanding of LOY's biological and clinical significance in hematopoiesis and cancer. Thus, there is an urgent need for simpler, more sensitive detection methods to better understand LOY's role in cancer. Research into the effects of hematopoietic and cancer-associated LOY on cancer progression is still in its early stages. Initial findings indicate that both forms of LOY may negatively affect cancer outcomes, potentially by altering immune responses and making cancer cells less susceptible to immune system attacks. Cancer-associated mLOY and hematopoietic mLOY may exacerbate cancer prognosis, possibly by making cancer cells more resistant to PD-1 ICB therapy. Targeting Y chromosome genes like UTY and KDM5D to improve sensitivity to PD-1 ICB therapy in non-mLOY cancers could become a new treatment approach. LOY-positive tumors show reduced response to anti-PD-1 therapy, indicating treatment insensitivity. Men with lower Y chromosome gene expression levels generally benefit more from radiotherapy. In conclusion, Y chromosome genes critically influence immune responses and survival rates [142].

Thus, XCI escape and LOY contribute to sex differences in immunity. X-linked genes and X-linked immune-related miRNAs enhance immune responses in females, affecting autoimmune disease susceptibility and cancer progression. Investigating these genes offers potential targets for personalized therapies. Meanwhile, LOY in immune cells is associated with increased mortality and susceptibility to diseases, particularly cancer, through its impact on immune responses and immune evasion. These mechanisms highlight the importance of XCI escape and LOY in immune cell function and disease, offering new opportunities for sex-specific diagnostics and therapies.

## Conclusion

Historically, sex differences in health and disease have been ignored or marginalized. Increasing evidence shows marked variations in disease manifestations and immune responses between males and females, with sex chromosomes playing a key role in these disparities. Sex chromosomes are crucial in innate and adaptive immune responses, leading to sex-specific differences in infectious diseases, autoimmune disorders, cancers, and vaccine efficacy. This review examines how abnormal expression of sex chromosome-linked genes affects sex differences in immune responses, focusing on mechanisms like XCI escape and LOY. This article aims to clarify how sex chromosomes influence immune response differences and disease-specific sex disparities, and to identify potential therapeutic targets. However, most existing studies on sex chromosomes' impact on immune-disease differences rely on case studies and lack depth, with many biological investigations failing to integrate sex chromosome factors comprehensively. Assessing sex-specific dosing through clinical trials is essential to optimize the right balance between drug effectiveness and toxicity, especially for medications with notable pharmacokinetic variations. Thus, it is essential to thoroughly examine and address sex differences in disease across basic, translational, and clinical research. A deeper investigation into the specific molecular mechanisms by which sex chromosomes drive sex differences in immune responses and disease is essential for achieving personalized and precise medical care.

## Supplementary Information

Supplementary Material 1.

## Data Availability

Not applicable.

## Abbreviations

XCI: X chromosome inactivation
LOY: Loss of Y chromosome
SLE: Systemic lupus erythematosus
HIV-1: Human Immunodeficiency Virus 1
Treg cells: Regulatory T cells
PBMCs: Peripheral blood mononuclear cells
PBC: Primary biliary cholangitis
VAT: Visceral adipose tissue
GBM: Glioblastoma
HCC: Hepatocellular carcinoma
HPSCs: Human embryonic stem cells
CRC: Colorectal cancer
AML: Acute myeloid leukemia
