# Supplementary material for: Sex differences in disease: sex chromosome and immunity
Source: J Transl Med. 2024 Dec 27;22:1150. doi: 10.1186/s12967-024-05990-2 (PMC11673612; doi:10.1186/s12967-024-05990-2)
Supplement: Supplementary file 1 — Supplementary Material 1. [file 12967_2024_5990_MOESM1_ESM.docx]

**Table S1.** Human X-linked immune-related genes and escape from X Chromosome Inactivation (XCI).

| **Gene ID** | **Gene names** | **Located** | **Known or potential role in immune** |
| --- | --- | --- | --- |
| 64109 | CRLF2 | Xp22.33 | This gene encodes a receptor for thymic stromal lymphopoietin, activating STAT3, STAT5, and JAK2 pathways, which control processes such as cell proliferation and hematopoietic system development. |
| 1438 | CSF2RA | Xp22.1 | This gene encodes the alpha subunit of the heterodimeric receptor for colony stimulating factor 2, controlling the production, differentiation, and function of granulocytes and macrophages. |
| 3563 | IL3RA | Xq24 | This gene encodes a subunit of a heterodimeric cytokine receptor that binds IL3. The receptor includes a ligand-specific α subunit and a signal-transducing β subunit shared with IL3, CSF2/GM-CSF, and IL5 receptors. |
| 8233 | ZRSR2 | Xp22.2 | This gene encodes a splicing factor that binds to the U2 auxiliary factor heterodimer to recognize functional 3' splice sites during pre-mRNA splicing. By influencing biological processes such as signal transduction, cell cycle, and apoptosis in immune cells, it regulates their function. Mutations in the ZRSR2 gene have been associated with hematological disorders such as myelodysplastic syndromes and acute myeloid leukemia . |
| 51284 | TLR7 | Xq28 | The gene encodes a member of the Toll-like receptor family, TLR7, which is closely associated with interactions with immune cells such as B cells, dendritic cells, and T cells. It regulates the direction and strength of the immune response by influencing cytokine expression and the balance of cell subsets. |
| 51311 | TLR8 | Xp22.2 | TLR8 significantly impacts immune cells by enhancing the function of regulatory T cells and glucose metabolism in cancer therapies, and its heightened expression in plasmacytoid dendritic cells and macrophages is linked to autoimmune conditions. In females, TLR8's presence on both X chromosomes is crucial for the production of IgG autoantibodies, type I IFN expression, and neutrophil generation. |
| 50943 | FOXP3 | Xp11 | FOXP3 regulates immune functions, particularly in the development and function of Treg cells, with its expression being controlled by the IL-2/Jak/Stat pathway. Gender differences affect FOXP3 expression and function, with variations in Treg cell counts and roles observed in diseases such as SSc and melanoma. |
| 3476 | IGBP1 | Xq13.1 | IGBP1 serves as a pivotal adaptor protein within the BCR signaling pathway. It interacts with the BCR complex, facilitating the transmission of signals from BCR to downstream signaling pathways. This process is crucial for initiating the tyrosine phosphorylation reaction upon antigen binding to BCR, thereby activating multiple signal transduction routes that are vital for B-cell proliferation and differentiation. |
| 8473 | OGT | Xq13.1 | OGT regulates cellular processes by catalyzing O-GlcNAc modifications, which are crucial for the activation of T cells and B cells. Lower levels of Ogt result in decreased expression of the activation marker CD69, thereby impacting immune responses. In female SLE patients, higher expression of Ogt and hypomethylation of its promoter in CD4+ T cells have been linked to sustained lymphocyte activation in SLE. |
| 2833 | CXCR3 | Xq13 | A G protein-coupled receptor with selectivity for three chemokines, termed CXCL9, CXCL10 and CXCL11. Binding of chemokines to this protein induces cellular responses that are involved in leukocyte traffic, most notably integrin activation, cytoskeletal changes and chemotactic migration. |
| 695 | BTK | Xq22.1 | The protein encoded by the BTK gene plays a pivotal role in B-cell development. Mutations in this gene can lead to X-linked agammaglobulinemia type 1, an immunodeficiency characterized by the inability to produce mature B lymphocytes and associated with a failure of Ig heavy chain rearrangement. |
| 3597 | IL13RA1 | Xq24 | Serves as a primary IL13-binding subunit of the IL13 receptor, and may also be a component of IL4 receptors. Binds to TYK2, which lead to the activation JAk1, STST3 and STAT6 induced by IL13 and IL4. |
| 4068 | SH2D1A | Xq25 | The protein encoded by the SH2D1A gene plays a major role in the bidirectional stimulation of T and B cells. SH2D1A interacts with other related surface molecules expressed on activated T cells, B cells, and NK cells, thereby modifying the signal transduction pathways in these cells. |
| 54440 | SASH3 | Xq26.1 | The SH3 protein contains a Src homology 3 domain and a sterile alpha motif, both of which are found in proteins involved in cell signaling. The SH3 protein may function as a signaling adapter protein in lymphocytes. |
| 2000 | ELF4 | Xq26.1 | ELF4 is a transcriptional activator that can bind and activate the promoters of the CSF2, IL3, IL8, and PRF1 genes. ELF4 is involved in the development and function of natural killer cells, innate immunity, and the induction of cell cycle arrest in naive CD8+ cells. |
| 959 | CD40LG | Xq26.3 | The CD40LG protein is expressed on the surface of T cells and regulates B cell function by binding to CD40 on the surface of B cells. A defect in this gene results in an inability to undergo immunoglobulin class switch and is associated with hyper-IgM syndrome. |
| 3654 | IRAK1 | Xq28 | IRAK1 binds to IL1R upon stimulation and is partially responsible for the upregulation of the transcription factor NF-kappa B induced by IL1. |
| 8517 | IKBKG | Xq28 | The IKBKG gene encodes the regulatory subunit of the inhibitor of kappaB kinase (IKK) complex, which activates NF-kappaB, leading to the activation of genes involved in inflammation, immunity, cell survival, and other pathways. |
| 4515 | MTCP1 | Xq28 | The MTCP1 gene is involved in some t(X;14) translocations associated with mature T-cell proliferations and may be implicated in leukemogenesis. |
| 3581 | IL9R | Xq28 | The IL9R gene encodes a cytokine receptor that specifically mediates the biological effects of interleukin 9 (IL9). IL9R forms a complex with the interleukin 2 receptor gamma (IL2RG). Ligand binding to this receptor leads to the activation of various JAK kinases and STAT proteins, which are connected to different biological responses. |
| 3920 | LAMP2 | Xq24 | LAMP2 plays a crucial role in the immune system by participating in lysosome function, which is essential for antigen processing and presentation. Additionally, it may influence tumor cell metastasis and immune responses by interacting with selectins and affecting cell adhesion. |
| 389856 | USP27X | Xp11.23 | USP27X is involved in immune regulation by modulating the levels of Bim, a pro-apoptotic protein that influences cell death and survival. By increasing Bim levels, USP27X may help counteract anti-apoptotic signals in immune cells and potentially impact immune responses and tumor suppression. |
| 331 | XIAP | Xq25 | The XIAP belongs to the apoptotic suppressor protein family. XIAP exerts its function by binding to tumor necrosis factor receptor-associated factors TRAF1 and TRAF2, inhibiting apoptosis induced by menadione, a potent free radical inducer, and interleukin 1-beta converting enzyme. |
| 1654 | DDX3X | Xp11.4 | DDX3X influences immune responses by modulating the regulation of mRNA processing and translation, which affects the expression of immune-related genes. Additionally, it may play a role in cellular signaling pathways that impact immune cell function and viral infections. |
| 7503 | XIST | Xq13.2 | DXIST, a long non-coding RNA, is involved in X chromosome inactivation and may influence immune system function by modulating gene expression related to immune responses. Disruptions in DXIST expression can potentially impact the balance of immune cell function and contribute to autoimmune disorders. |
| 1756 | DMD | Xp21.2-p21.1 | DMD is crucial for maintaining muscle cell integrity, and its loss or dysfunction can lead to chronic inflammation and immune system activation in dystrophic muscles. The resulting inflammatory environment can further exacerbate muscle damage and contribute to the progression of Duchenne muscular dystrophy. |
| 5613 | PRKX | Xp22.33 | PRKX may influence immune responses through its role in cellular signaling and development, potentially affecting the maturation of immune cells such as macrophages and granulocytes. Abnormalities in PRKX function can disrupt these processes, potentially impacting immune system regulation and contributing to disorders involving immune cell dysfunction. |
| 5009 | OTC | Xp11.4 | OTC is essential for detoxifying ammonia in the urea cycle, and its deficiency can lead to high ammonia levels that affect multiple organ systems, including the immune system. Elevated ammonia levels can disrupt immune cell function and contribute to immune system dysregulation and related disorders. |
| 80231 | CXorf21 | Xp21 | CXorf21 is involved in the positive regulation of innate immune responses, positively regulates the Toll-like receptor signaling pathway, and regulates the pH of the lysosomal lumen. |
| 8269 | TMEM187 | Xq28 | TMEM187 may influence immune responses by regulating the function of membrane proteins involved in immune cell signaling and function. Abnormal expression or dysfunction of TMEM187 could disrupt normal immune system operation and contribute to immune-related disorders. |
| 1536 | CYBB | Xp21.1-p11.4 | CYBB is a key component of the microbial killing oxidase system in phagocytes, involved in generating reactive oxygen species to kill bacteria. Deficiency of CYBB leads to chronic granulomatous disease, where neutrophils can engulf bacteria but fail to kill them due to insufficient production of reactive oxygen, resulting in impaired infection resistance. |
| 7403 | KDM6A | Xp11.3 | KDM6A plays a role in immune regulation by modifying histone methylation patterns, which can influence the expression of genes involved in immune responses. Dysregulation of KDM6A can affect immune cell differentiation and function, potentially contributing to autoimmune disorders and cancer. |
| 8242 | KDM5C | Xp11.22 | KDM5C is involved in regulating gene expression through histone modification, impacting immune cell differentiation and function. Dysregulation of KDM5C can lead to altered immune responses and has been linked to X-linked cognitive disabilities and potentially to immune-related disorders. |
| 7454 | WAS | Xp11.23 | WAS is involved in transmitting signals from cell surface receptors to the actin cytoskeleton. Wiskott-Aldrich syndrome is a rare, inherited X-linked recessive disease characterized by immune dysregulation and microthrombocytopenia. The WAS gene product is a cytoplasmic protein expressed exclusively in hematopoietic cells. |
| 6197 | RPS6KA3 | Xp22.12 | RPS6KA3 is involved in the regulation of various immune cell functions by phosphorylating components of the MAPK signaling pathway. Dysregulation of RPS6KA3 can affect immune cell growth and differentiation, potentially contributing to immune-related disorders and impacting overall immune responses. |
| 8227 | AKAP17A | Xq28 | AKAP17A is involved in the regulation of alternative splicing of mRNA precursors, which can affect the expression of immune-related genes. Its role in the spliceosome complex may influence immune cell function and contribute to the modulation of immune responses. |
| 401577 | CD99P1 | Xp11.3 | CD99P1 is involved in regulating cell adhesion and signaling, which can influence immune cell migration and interactions. Dysregulation of CD99P1 may impact immune responses and contribute to the development of autoimmune diseases or other immune system disorders. |
| 5277 | PIGA | Xq28 | PIGA is essential for synthesizing the GPI anchor, which is crucial for attaching various cell surface proteins involved in immune responses. Mutations in PIGA can lead to paroxysmal nocturnal hemoglobinuria (PNH), a condition that affects immune cell function and can result in increased susceptibility to infections and other immune-related issues. |
| 369 | ARAF | Xp11.3 | ARAF is involved in regulating the TOR signaling pathway, which affects various immune cell functions, including cell growth and survival. Dysregulation of ARAF can impact immune responses and is associated with the development of certain cancers, including high-grade gliomas, which can influence overall immune system health. |
| 5199 | CFP | Xp11.23 | CFP positively regulates the alternative complement pathway, enhancing the immune system's ability to target and eliminate pathogens. Deficiencies in CFP can lead to increased susceptibility to infections, particularly meningococcal infections, due to impaired formation of the membrane attack complex. |
| 139716 | GAB3 | Xq28 | GAB3 is involved in the signaling pathways of growth factors and cytokines, playing a key role in macrophage differentiation and immune cell activation. Its function as a scaffolding protein supports the organization and amplification of immune responses by interacting with signaling molecules like SHP2 and GRB2. |
